# PANDEMIC RECOVERY IN NURSING HOMES: IMPACT OF A 5-WEEK CURRICULUM ON RESILIENCE AND GROWTH IN DIRECT CARE WORKERS

**DOI:** 10.1093/geroni/igac059.2975

**Published:** 2022-12-20

**Authors:** Susan Wehry, Prakash Patel

**Affiliations:** University of New England, Biddeford, Maine, United States; University of New England College of Osteopathic Medicine, Biddeford, Maine, United States

## Abstract

The COVID-19 pandemic significantly impacted people working and living in nursing homes. A virtual five-week curriculum was developed to support recovery and foster growth in direct care workers (DCWs). We hypothesized DCWs would be interested, acquire knowledge and self-care skills, and utilize a post traumatic growth (PTG) self-assessment tool for reflection. The 5 week voluntary program was offered twice. Sessions consisted of a didactic presentation, Q&A, skill practice and action commitment. Each 30 minute session was offered twice weekly, a.m. and p.m., to accommodate shift schedules. Polls were conducted within sessions. Participants who attended all sessions received a certificate of attendance and completed a 4 question, 5-point Likert scale survey and the PTG tool. 53 DCWs participated in at least one session; 30 completed the program and ranked weeks 2 (95%), 3 (65%), and 4 (60%) as most useful. Most used skills were breathing (95%), gratitude practice (91%), catch-check-change (61%), self-massage (61%), and reducing exposure to toxic news (61%). DCWs endorsed self-improvement (96%), curiosity (91%), and “having a hard time” (61%) as most common reasons for enrolling. 100% reported the course completely or almost completely met their goals. In-session polling indicated attendees “felt better or more confident” at the conclusion of each session. 100% successfully utilized the PTG tool. The curriculum was responsive to DCWs needs and interests. DCWs acquired and utilized resilience skills. Some components of the curriculum were more effective than others. Shorter staff training sessions are more attractive to staff and employers. Further study is warranted.

